# Endogenous Protease Nexin-1 Protects against Cerebral Ischemia

**DOI:** 10.3390/ijms140816719

**Published:** 2013-08-14

**Authors:** Osvaldo Mirante, Melanie Price, Wilfredo Puentes, Ximena Castillo, Corinne Benakis, Jonathan Thevenet, Denis Monard, Lorenz Hirt

**Affiliations:** 1 Stroke Laboratory, Neurology Service, Department of Clinical Neurosciences, Centre Hospitalier Universitaire Vaudois and Lausanne University, Lausanne 1011, Switzerland; E-Mails: mirante@hifo.uzh.ch (O.M.); melprice_ch@yahoo.com (M.P.); wpuentes@outlook.com (W.P.); ximena.Castillo@unil.ch (X.C.); Corinne.Benakis@unil.ch (C.B.); jonathanthevenet@yahoo.fr (J.T.); 2 Friedrich Miescher Institute for Biomedical Research, Basel 4058, Switzerland; E-Mail: denis.monard@fmi.ch

**Keywords:** protease nexin-1, thrombin preconditioning, cerebral ischemia, organotypic hippocampal slice cultures, glucose and oxygen deprivation, c-Jun *N*-terminal kinase, stroke, neuroprotection

## Abstract

The serine protease thrombin plays a role in signalling ischemic neuronal death in the brain. Paradoxically, endogenous neuroprotective mechanisms can be triggered by preconditioning with thrombin (thrombin preconditioning, TPC), leading to tolerance to cerebral ischemia. Here we studied the role of thrombin’s endogenous potent inhibitor, protease nexin-1 (PN-1), in ischemia and in tolerance to cerebral ischemia induced by TPC. Cerebral ischemia was modelled *in vitro* in organotypic hippocampal slice cultures from rats or genetically engineered mice lacking PN-1 or with the reporter gene lacZ knocked into the PN-1 locus PN-1HAPN-1-lacZ/HAPN-1-lacZ (PN-1 KI) exposed to oxygen and glucose deprivation (OGD). We observed increased thrombin enzyme activity in culture homogenates 24 h after OGD. Lack of PN-1 increased neuronal death in the CA1, suggesting that endogenous PN-1 inhibits thrombin-induced neuronal damage after ischemia. OGD enhanced β-galactosidase activity, reflecting PN-1 expression, at one and 24 h, most strikingly in the stratum radiatum, a glial cell layer adjacent to the CA1 layer of ischemia sensitive neurons. TPC, 24 h before OGD, additionally increased PN-1 expression 1 h after OGD, compared to OGD alone. TPC failed to induce tolerance in cultures from PN-1^−/−^ mice confirming PN-1 as an important TPC target. PN-1 upregulation after TPC was blocked by the c-Jun *N*-terminal kinase (JNK) inhibitor, L-JNKI1, known to block TPC. This work suggests that PN-1 is an endogenous neuroprotectant in cerebral ischemia and a potential target for neuroprotection.

## 1. Introduction

Stroke is a leading cause of mortality and disability worldwide and results mostly from an ischemia of part of the brain, triggering cell death pathways which lead to both rapid and delayed injury to the brain parenchyma. Thrombolysis within 4.5 h from symptom onset, the only currently available specific treatment for ischemic stroke, can be administered to only approximately 10% of patients hence the need for alternative treatments such as neuroprotective approaches [1]. Tolerance to ischemia can be elicited by exposure to a low intensity noxious stimulus (preconditioning) [2]. The tolerance to subsequent ischemia induced by the administration of a low dose of the serine protease thrombin [3–5] may provide opportunities to identify some of the molecules involved in endogenous neuroprotective mechanisms [6]. This phenomenon seems a paradox given that thrombin extravasation is considered as an important cause of brain cell damage and high doses of thrombin injected directly into the brain elicit oedema and neurotoxicity [7,8]. Recently, an *in vivo* study using an ACPP (activated cell penetrating peptide) imaging probe to detect the proteolytic activity of thrombin, showed that early activation of thrombin during acute focal cerebral ischemia strongly correlated with neurovascular damage in the ischemic core [9]. Thrombin can be inhibited by serine protease inhibitors, such as antithrombin III or protease nexin-1 (PN-1). PN-1 is a glia-derived secreted 43 kD protein, member of the serpin family, with neurite outgrowth promoting activity [10]. It is a very potent inhibitor of thrombin and also inhibits Factor Xa, Factor XIa, tPA, uPA and plasmins. It is expressed in many organs including the brain and vessel walls. PN-1 is currently considered an important regulator of thrombin in both the intra- and extravascular space (for review [11]).

Thrombin induces both tolerance and cell death through proteolytic cleavage of the protease-activated receptor-1 (PAR-1), with the GTPase RhoA being involved in both cases [4]. Low concentrations (0.01 U/mL) of mouse thrombin added to organotypic hippocampal slices, 24 h before OGD, attenuate death of CA1 neurons [5]. It has been shown that these neuroprotective thrombin concentrations induce Ca^2+^ spikes, while higher, toxic concentrations induce sustained Ca^2+^ entry into CA1 neurons of hippocampal slices [12]. *In vivo*, intracerebroventricular (i.c.v.) injection of 0.01 U of mouse thrombin, 24 h before 30 min middle cerebral artery occlusion (MCAO) in mice, reduces the infarct volume and improves the behavioural outcome [5]. In rat models, TPC requires activation of the extracellular signal-regulated kinases 1 and 2 (ERK1/2) pathways [13,14]. The ERK1/2 inhibitor, PD98059, prevents the protective effect. TPC also increases hypoxia inducible factor-1α [15] and heat shock proteins proposed as putative downstream effectors controlling the extent of damage [3].

On the contrary, strong protection is achieved by intra-cerebral injection of hirudin, a potent thrombin inhibitor from leech, before rat MCAO [16] or global cerebral ischemia in gerbils [12]. In blood free organotypic rat hippocampal slice cultures, ischemia which is modelled by OGD, triggers the generation of thrombin [17]. Both hirudin and PN-1 lead to neuroprotection when added after OGD demonstrating a role of thrombin signalling in ischemic damage in neural tissue. Thrombin thereby appears to play a dual paradoxical role in cerebral ischemia. Interestingly, the inhibition of the c-Jun *N*-terminal kinase (JNK) prevents the neuroprotection induced by TPC [5]. As JNK inhibition strongly protects in rodent models of cerebral ischemia [18–20], JNK appears to have a similar dual function.

PN-1 expression is increased after global ischemia [21,22] and after intracerebral haemorrhage [23]. This serpin is therefore likely to play a key role as regulator of thrombin activity following brain injury. Consequently, we investigated its function in cerebral ischemia using newly available genetically modified mice: PN-1 knockout mice and knock-in animals expressing a reporter gene in the PN-1 locus [24,25]. Our results indicate that PN-1 may be involved in the endogenous defence mechanisms triggered by TPC.

## 2. Results and Discussion

### 2.1. Thrombin Activity Is Increased in Brain Tissue after Ischemia *in Vitro*

**Figure 1:**
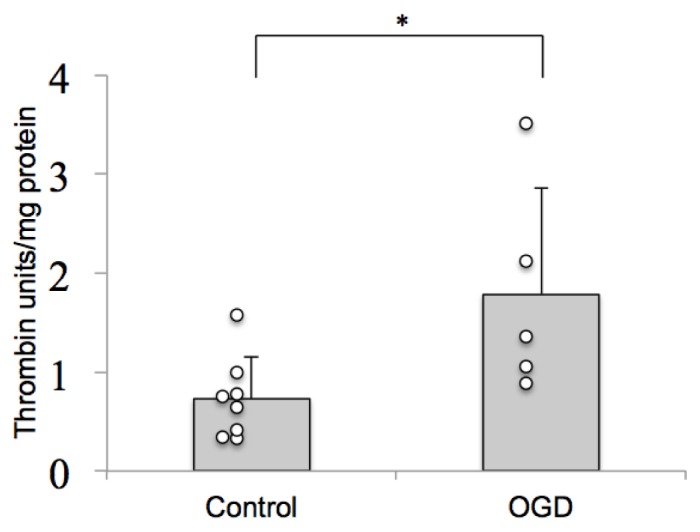
Thrombin activity is increased after OGD. Thrombin enzyme activity in hippocampal slice culture homogenates was assayed using a chromogenic substrate with purified recombinant mouse thrombin as a standard. The results were expressed as units of thrombin/mg of protein. Thrombin enzyme activity is significantly (Kruskall Wallis test *p* = 0.0006, followed by Mann-Whitney test *p* < 0.05) increased in hippocampal slice cultures 24 h after oxygen and glucose deprivation (OGD). (* *p* < 0.05).

Thrombin appears to play a role as a mediator of ischemic neuronal death in the cerebral parenchyma. Prothrombin was detected in organotypic hippocampal slice cultures by Western blotting and was shown to be converted to thrombin in response to OGD [17]. We tested whether this increased thrombin immunoreactivity, after a 30 min OGD followed by 24 h reoxygenation, corresponds to increased thrombin protease activity in the hippocampal slices. Using a chromogenic substrate for thrombin, we evaluated thrombin protease activity in hippocampal slice culture homogenates and compared it to the activity of purified mouse thrombin. 24 h after OGD, there was a significant increase in thrombin activity, demonstrating activation of thrombin in response to ischemia (Figure 1). This thrombin protease activity, produced in an *in vitro* “blood-free” system correlates with neuronal cell death observed in the slices 24 h after OGD.

### 2.2. Endogenous PN-1 Protects against Ischemic Neuronal Death in Hippocampal Slices after OGD

**Figure 2:**
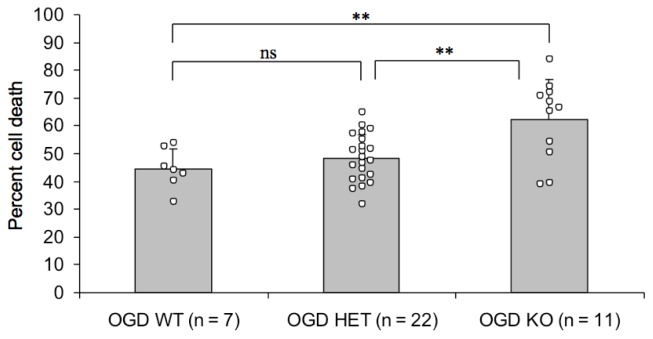
Enhanced sensitivity to OGD in the absence of PN-1. The role of PN-1 in cerebral ischemia was evaluated by subjecting mouse hippocampal slice cultures to OGD and measuring neuronal cell death in the CA1 by propidium iodide uptake. Mutant mice lacking one allele of PN-1 (heterozygotes, HET) were not significantly different from wild type mice (WT). In PN-1 knockout mice (KO) however, the amount of cell death was significantly higher than in wild types or heterozygotes (one-way ANOVA followed by Tukey-Kramer, *p* < 0.01). N represents the number of wells containing 3 to 5 slices each. (** *p* < 0.01).

As the addition of recombinant PN-1 after OGD attenuates ischemic neuronal death [17], we investigated the role of endogenous PN-1 in cerebral ischemia by subjecting hippocampal slices from mice lacking PN-1 [24] to OGD. Neuronal death 48 h after OGD was significantly higher in slices from mice lacking PN-1 than from control mice, with intermediate results in heterozygotes, demonstrating that endogenous PN-1 has a protective role, most likely by attenuating the detrimental action of thrombin (Figure 2).

### 2.3. PN-1 Is Upregulated after OGD and Thrombin Preconditioning

**Figure 3:**
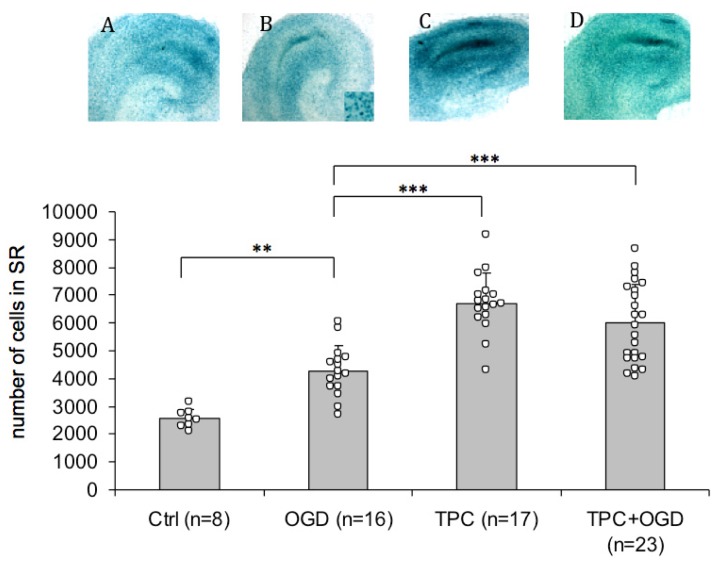
TPC enhances PN-1 transcription *in vitro.* Organotypic hippocampal slice cultures from PN-1 KI mice expressing the β-galactosidase protein under the control of the PN-1 promoter were used. X-gal staining reflecting β-galactosidase activity was detected and quantitated in the stratum radiatum. The stratum radiatum was identified by double labellling with X-gal and the neuronal marker Neu N, which labels the neuronal layers. The major site of X-Gal labelling did not coincide with Neu N labelling suggesting glial cell labelling. Compared to controls (**A**), OGD (**B**) significantly enhanced PN-1 expression. TPC alone (**C**) further enhanced the expression of PN-1 (ANOVA followed by Tukey-Cramer, *p* < 0.001, compared to OGD alone). If OGD was preceded by TPC, (**D**), the expression was significantly higher than with OGD alone (ANOVA followed by Tukey-Cramer, *p* < 0.001). N represents the total number of slices. A high magnification image of X-gal stained cells is inserted into the right-hand corner of image B. (** *p* < 0.01; *** *p* < 0.001).

As thrombin can induce tolerance to ischemia [6], PN-1 may be a potential key player in thrombin preconditioning, which could result in an enhanced PN-1 expression. To test this possibility, we used knock-in mice (PN-1 KI) expressing the β-galactosidase reporter gene under the control of the PN-1 promoter [25]. The presence of β-galactosidase, reflecting PN-1 transcription, was revealed in hippocampal slices by X-gal staining (Figure 3, panels A to D). The staining was most prominent in the stratum radiatum, a glial cell region in the vicinity of the ischemia sensitive CA1 neurons. The expression of β-galactosidase was significantly enhanced both 1 h (control *vs.* OGD, Figure 3) and 24 h after OGD onset (not shown). Having shown previously that thrombin signalling after OGD occurs rapidly, as hirudin administered immediately after OGD for 1 h conferred the same protection as hirudin applied for 24 h after OGD [17], we focused on the 1 h time-point. TPC alone further significantly enhanced the expression of PN-1 in the stratum radiatum compared to OGD alone (TPC *vs.* OGD, Figure 3), as did TPC 24 h before OGD (TPC+OGD *vs.* OGD, Figure 3). Similar results were obtained by performing β-galactosidase assays on slice culture homogenates (data not shown). Increased PN-1 protein is observed on Western blots 1 h and 24 h after OGD and after TPC in a model system using rat hippocampal slices subjected to OGD. Comparable results were observed for TPC when using thrombin receptor agonist or thrombin, indicating that upregulation of PN-1 is via the PAR-1 receptor (data not shown and [6]). Also, we observed qualitative increases in PN-1 transcription *in vivo* in KI mice after injecting preconditioning doses of thrombin into the lateral ventricles and after MCAO modeled ischemia (data not shown).

The upregulation of endogenous PN-1 in response to ischemia and to thrombin preconditioning confirms a role for the serpin as a regulator of thrombin activity after ischemia and in TPC and suggests that PN-1 has a neuroprotective role in OGD.

### 2.4. Endogenous PN-1 Is Necessary for Thrombin Induced Ischemic Tolerance in Hippocampal Slice Cultures

**Figure 4:**
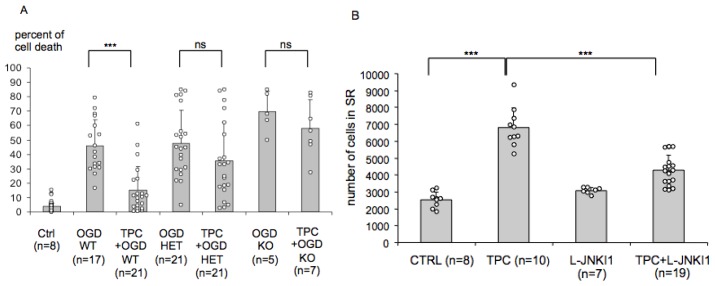
TPC requires the presence of PN-1. (**A**) TPC was performed in slices from mice lacking PN-1. TPC before OGD significantly (*p* < 0.001, Kruskall Wallis followed by Dunn’s multiple comparisons) attenuated neuronal death in cultures from wild-type mice. TPC did not induce a significant protection (ns) in heterozygotes or knock-out mice, demonstrating that endogenous PN-1 is required for TPC. N represents the number of slices. (*** *p* < 0.001); (**B**) Given the identification of PN-1 as a target for TPC, slices from PN-1 KI mice were used to test whether the TPC-induced upregulation of PN-1 was influenced by the JNK inhibitor L-JNKI1, known to block the protective effect of TPC. β-galactosidase positive cells in the stratum radiatum were counted. TPC enhanced the expression of PN-1 (ANOVA *p* < 0.0001, Tukey-Cramer *p* < 0.001) while L-JNKI1 alone did not. The addition of L-JNKI1 after TPC significantly reduced the PN-1 upregulation (ANOVA *p* < 0.0001, Tukey-Cramer *p* < 0.001 compared to TPC alone). (*** *p* < 0.001).

To further evaluate the importance of the observed changes, we tested whether cultures from PN-1 knock-out mice could be preconditioned by thrombin *in vitro* (Figure 4A). While tissue cultures from wild type mice were protected by exposure to 0.01 U/mL of mouse thrombin 24 h before OGD (very significant 67% reduction in neuronal death), we did not observe a protection in heterozygotes (non-significant 26% reduction in neuronal death) or in knock-outs (non-significant 16% reduction in neuronal death). This suggests that endogenous PN-1 is necessary for the induction of tolerance by thrombin.

### 2.5. PN-1 Upregulation in Hippocampal Slices Is Prevented by Inhibiting the c-Jun-*N*-Terminal Kinase (JNK)

*In vivo* L-JNKI1, a cell permeable peptide inhibitor of JNK, prevents TPC [5]. *In vitro*, both L-JNKI1 and the small molecule JNK inhibitor SP600125 prevented thrombin-induced tolerance [5,6]. Having identified PN-1 as a target for TPC we tested the effect of L-JNKI1 on the upregulation of PN-1 in PN-1 KI mice. While L-JNKI1 alone had no effect on the baseline expression of PN-1, it blocked the very significant 2.6 fold PN-1 upregulation after thrombin preconditioning (Figure 4B), further supporting a role for PN-1 in thrombin-induced tolerance to ischemia.

### 2.6. Discussion

The protease-activated receptors, the serpin PN-1 and Factor X, main players involved in thrombin signalling and regulation are detected in the brain [10,26–29] and increasing evidence indicates that the clotting factor thrombin plays a role in the cerebral parenchyma (for review [30]). Using a blood free *in vitro* approach, we previously showed that thrombin protein is detected on Western blots after ischemia modelled *in vitro* by OGD and that specific thrombin and Factor Xa inhibitors confer neuroprotection [17,31]. The results presented here make two further points:

Firstly, we demonstrate an increase in thrombin protease enzyme activity in brain tissue after ischemia (Figure 1). This suggests that thrombin may be activated in an *in vitro* system, free of circulating blood.

Secondly, our observation that CA1 hippocampal neurons from PN-1 knockout mice are significantly more susceptible to ischemia (Figure 2) provides strong support for an important function of PN-1. It certainly implies that PN-1 can to some extent moderate the deleterious effect of the ischemia-induced thrombin activity on neuronal death in wild type mice.

To further investigate this role of PN-1 in the CNS, we tested cultures from PN-1 knock-in mice in which the expression of bacterial β-galactosidase is driven by the PN-1 promoter [25]. While PN-1 is secreted, the reporter protein β-galactosidase bears a nuclear localisation signal that remains in the nucleus of the cell, thus identifying the site of PN-1 transcription. Detection of the activity of the reporter enzyme in homogenates also provides information on the level of expression. We observed that β-galactosidase was strongly expressed in hippocampal cultures and the site of highest transcription is a glial cell layer, the stratum radiatum, in close vicinity to the pyramidal cell layer of the CA1, where most ischemia sensitive neurons reside. A significant increase in β-galactosidase activity in the stratum radiatum was detected both after ischemia and TPC (Figure 3). Furthermore, TPC, followed 24 h later by OGD, induced a significantly higher β-galactosidase activity than OGD alone (Figure 3) This suggests that OGD triggers the expression of PN-1 in an attempt to counteract the detrimental increase of thrombin activity and that TPC further enhances this protective mechanism. β-galactosidase expression is upregulated *in vivo* after i.c.v. injection of preconditioning doses of thrombin and after MCAO (data not shown). Interestingly, the highest PN-1 expression is in cells lining the ventricles which coincides with thrombin receptor PAR-1 expression [32] and therefore thrombin, in the ventricles, could trigger the expression of its inhibitor via its receptor in these cells, thereby modulating its own activity.

We performed MCAO experiments on PN-1^−/−^ mice and observed a tendency to slightly larger lesions in PN-1^−/−^ mice compared to WT although results were not significant and highly variable between experimenters (data not shown). Similarly, there was a trend for a worse behavioural outcome in the PN-1^−/−^ mice but again this did not reach significance (data not shown). This may be due to compensatory mechanisms in PN-1 knock-out mice and indeed, Hengst *et al*. [33] commented that mice lacking PN-1 show only very subtle phenotypes in the CNS and went on to purify and characterize a novel serine protease inhibitor, phosphatidylethanolamine-binding protein from the brains of PN-1^−/−^ mice which specifically interferes with thrombin activity. In this respect it will be useful to knock-down the activity of PN-1 at selected time points in the mouse brain to investigate the role of PN-1 in the absence of possible compensatory mechanisms. As thrombin proteolytic activity is also associated with long-term cognitive deficit [9] it will be interesting to study PN-1 knock-down and possible behavioural differences at late time points. Cultures from PN-1 deficient mice and heterozygotes with a single PN-1 allele failed to develop significant protection after TPC while TPC induced significant protection in slices from wild type littermates (Figure 4A). Endogenous PN-1 is required for tolerance induced by TPC, and may therefore be an important target for TPC. Indeed, administration of L-JNKI1 [5,6,34] after TPC prevented the enhanced PN-1 expression (Figure 4B), providing an additional argument for an important role of PN-1 in TPC.

High doses of thrombin injected into the brain trigger cerebral oedema and it is also known that the oedema occurring after intracerebral haemorrhage (ICH) is partly due to thrombin (for review [35]). Oedema is also an aggravating factor in cerebral ischemia. Taking into account these deleterious effects, it is important to better understand the protective impact of TPC, both upstream and downstream of thrombin, to characterise the molecules involved. We have identified a downstream target of TPC, the water channel aquaporin 4 (AQP4), which is upregulated on astrocytic endfeet 1 h after ischemia onset and which prevents the occurrence of early cerebral oedema [36,37]. This supports interplay between thrombin and AQP4. Others have shown that thrombin induces either cell death or protection in primary astrocytic cultures via PAR-1 activation of the small GTPase RhoA [4,38–40]. Thrombin triggers a protective mechanism via PAR-dependent astrocytic release of chemokine growth-regulated oncogene/cytokine-induced neutrophil chemoattractant-1 (GRO/CINC-1) [41–43]. Given our present demonstration that PN-1 is a necessary target in TPC, it will be interesting to study to what extent this serpin affects AQP4, PAR activation, small GTPase RhoA and GRO/CINC-1. A further exciting approach will be to use the presence or absence of PN-1 and other TPC targets to investigate at which point the thrombin pathway diverges to signal cell death or survival.

## 3. Experimental Section

### 3.1. Animals

All animal experiments were conducted in accordance with the European guidelines and were approved by the local authority (cantonal veterinary office). Wild type rats (OFA Sprague Dawley, Charles River, France) [20,44] and wild type mice (C57Bl6 Charles River, France) were used in our experiments. Mutant mice were obtained from the laboratory of Pr. Denis Monard, Friedrich-Miescher Institute, Basel: PN-1 knock-in mice with insertion of the β-galactosidase coding sequence behind the PN-1 coding sequence: PN-1HAPN-1-lacZ/HAPN-1-lacZ [25]; PN-1 knock-out mice, bred by crossing PN-1^+/−^ mice [24]). Heterozygous mating generated PN-1 KO and wild-type littermates. These PN-1 deficient mice are phenotypically normal apart from male infertility [45]. Genotyping was performed by PCR of DNA from tail biopsies [25].

### 3.2. Organotypic Hippocampal Slice Cultures

Organotypic hippocampal slice cultures were prepared as previously described from 10 to 12 day-old rats (OFA Sprague Dawley, Charles River, France) [20,44] or from 12-day old wild type (C57Bl6 Charles River, France) or mutant mice (PN-1 knock-in mice; PN-1 knock-out mice). Coronal hippocampal sections (350 μm) were placed onto sterile porous membrane units (Millicell-CM, Millipore, Billerica, MA, USA) in wells containing 1 mL of culture medium consisting of 50% MEM supplemented with HEPES and sodium bicarbonate, 25% Hank’s salt solution, 25% horse serum, 2 mM L-glutamine and 35 mM D-glucose. Cultures were kept at 33 °C, 100% humidity, 5% CO2 for 4 days, and then transferred to culture medium with 15% horse serum and 5 mM D-glucose, which was changed every 3 to 4 days. Experiments were started after 7 days of culture.

### 3.3. Oxygen and Glucose Deprivation (OGD) in Organotypic Hippocampal Slice Cultures

OGD experiments were performed as previously described in serum free hypoglycaemic DMEM medium (D5030, Sigma-Aldrich, St. Louis, MO, USA) supplemented with 1 mM D-glucose and 2 mM L-glutamine [17]. This medium was equilibrated for 1 h at 37 °C, in a humidified chamber (COY, Grass Lake, MI, USA) with a hypoxic atmosphere (5% O_2_, 5% CO_2_). Slices were transferred into this “buffered medium” and into the hypoxic chamber for 30 min. Control cultures were placed in DMEM medium supplemented with 5 mM D-glucose and 2 mM L-glutamine for 30 min at 37 °C, in a humid normoxic atmosphere. For recovery, cultures were transferred into culture medium at 33 °C for 24 or 48 h. For thrombin preconditioning, purified mouse thrombin (Sigma, final concentration, 0.01 U/mL) was added for 1 h, after which time the medium was changed, 24 h before OGD. Cell death was determined in the CA1 after incubating cultures (3–5 slices per culture) for 30 min with the fluorescent viability indicator propidium iodide (PI, 5 μg/mL). PI fluorescent emission (excitation wavelength 568 nm) was measured 24 h or 48 h after ischemia, using an epifluorescence microscope with a 5× lens coupled to a camera (Leica). PI images were acquired with standardized camera settings and optical density was measured with ImageJ software (ImageJ 1.36b, National Institute of Health). After subtracting the background fluorescence, results were expressed as a percentage of maximal cell death obtained by submerging slices in PBS for 24 h at 4 °C.

### 3.4. Chromogenic Thrombin Assay

Cultured hippocampal slices (5 slices in one microwell) were homogenised in 100 μL of homogenizing buffer (20 mM Sodium Phosphate, 0.32 M Sucrose, 1 mM EDTA, 0.2% Tween 20, in sterile water). Chromogenic assays were adapted to 96 well microtiter plates. The assay mixture (final volume 100 μL/well) included in addition to the samples, or to purified mouse thrombin (from Sigma) for standard curves, the following reagents (final concentrations): 67 mM Tris pH 8.0, 0.13% polyethylene glycol (PEG) 6000, 133 mM NaCl. The plate was shaken and 125 mg/mL chromogenic peptide S-2238 (S-2238TM *H*-D-Phenylalanyl-L-pipecolyl-L-arginine-*p*-nitroaniline dihydrochloride, stock solution: 1.2 mM in H_2_O, Chromogenix, Endothell, AG, Allschwil, Switzerland) was added. Plates were incubated at 37 °C in a humidified chamber and absorbance was measured at 405 nm in a Thermomax microplate reader [46]. To avoid contamination by exogenous thrombin activity from the horse serum-containing medium, hippocampal slice cultures were kept in serum free medium for 24 h before homogenisation.

### 3.5. β-Galactosidase Enzyme Staining on Hippocampal Slices

Hippocampal slices or frozen brain cryostat slices were fixed 30 min in 4% PFA/PBS, washed 3 × 15 min in washing buffer (0.01% NaDOC, 0.02% Nonidet P-40, 5 mM EGTA pH 8.4, 3 mM MgCl_2_, in PBS), and stained with an X-Gal staining solution 10 mM K_3_Fe(CN)_6_, 10 mM K_4_(Fe(CN)_6_)_2_, 0.25 mg/mL 5-Bromo-4-Chloro-3-Indolyl-b-galactopyranoside (X-Gal, Eppendorf, Germany, stock solution 50 mg/mL in Dimethylsulfoxide (DMSO)) during 3–8 h at 37 °C. Slices were then washed 5 min in PBS, fixed again in 4% PFA/PBS for 30 min, and finally washed 5 min in PBS. The Mercator program (Explora Nova, La Rochelle, France) was used to analyse the β-galactosidase staining on hippocampal slice cultures. The blue staining was analysed by computer assisted microscopic quantification of the number of positive cells in the stratum radiatum region of organotypic hippocampal slices.

### 3.6. Statistical Analysis

Data were expressed as mean ± SD. Statistical evaluation was performed using the Instat software (GraphPad, San Diego, CA, USA). In the case of two groups, data were compared with the Mann-Whitney test. Comparisons of 3 groups were carried out by ANOVA or by the Kruskall-Wallis test in cases of non parametrical data or small sample sizes. Probability values of <0.05 were considered significant.

## 4. Conclusions

Hippocampal slice cultures from Protease nexin-1 deficient mice are more vulnerable to experimental ischemia than cultures from wild type animals, indicating that PN-1 is an endogenous neuroprotectant. Furthermore, neuroprotection resulting from thrombin preconditioning is associated with an upregulation of PN-1 and TPC does not protect PN-1 deficient cultures, highlighting the role of PN-1 in tolerance to ischemia triggered by thrombin preconditioning. This work suggests that PN-1 is an endogenous neuroprotectant in cerebral ischemia and a potential target for neuroprotection.

## References

[1] Hacke W., Kaste M., Bluhmki E., Brozman M., Davalos A., Guidetti D., Larrue V., Lees K.R., Medeghri Z., Machnig T. (2008). Thrombolysis with alteplase 3 to 4.5 hours after acute ischemic stroke. N. Engl. J. Med.

[2] Dirnagl U., Simon R.P., Hallenbeck J.M. (2003). Ischemic tolerance and endogenous neuroprotection. Trends Neurosci.

[3] Xi G., Keep R.F., Hua Y., Xiang J., Hoff J.T. (1999). Attenuation of thrombin-induced brain edema by cerebral thrombin preconditioning. Stroke.

[4] Donovan F.M., Cunningham D.D. (1998). Signaling pathways involved in thrombin-induced cell protection. J. Biol. Chem.

[5] Granziera C., Thevenet J., Price M., Wiegler K., Magistretti P.J., Badaut J., Hirt L. (2007). Thrombin-induced ischemic tolerance is prevented by inhibiting c-jun *N*-terminal kinase. Brain Res.

[6] Price M., Badaut J., Thevenet J., Hirt L. (2010). Activation of c-Jun in the nuclei of neurons of the CA-1 in thrombin preconditioning occurs via PAR-1. J. Neurosci. Res.

[7] Lee K.R., Betz A.L., Keep R.F., Chenevert T.L., Kim S., Hoff J.T. (1995). Intracerebral infusion of thrombin as a cause of brain edema. J. Neurosurg.

[8] Xue M., del Bigio M.R. (2001). Acute tissue damage after injections of thrombin and plasmin into rat striatum. Stroke.

[9] Chen B., Friedman B., Whitney M.A., Winkle J.A., Lei I.F., Olson E.S., Cheng Q., Pereira B., Zhao L., Tsien R.Y. (2012). Thrombin activity associated with neuronal damage during acute focal ischemia. J. Neurosci.

[10] Gloor S., Odink K., Guenther J., Nick H., Monard D. (1986). A glia-derived neurite promoting factor with protease inhibitory activity belongs to the protease nexins. Cell.

[11] Bouton M.C., Boulaftali Y., Richard B., Arocas V., Michel J.B., Jandrot-Perrus M. (2012). Emerging role of serpinE2/protease nexin-1 in hemostasis and vascular biology. Blood.

[12] Striggow F., Riek M., Breder J., Henrich-Noack P., Reymann K.G., Reiser G. (2000). The protease thrombin is an endogenous mediator of hippocampal neuroprotection against ischemia at low concentrations but causes degeneration at high concentrations. Proc. Natl. Acad. Sci. USA.

[13] Xi G., Hua Y., Keep R.F., Duong H.K., Hoff J.T. (2001). Activation of p44/42 mitogen activated protein kinases in thrombin-induced brain tolerance. Brain Res.

[14] Jiang Y., Wu J., Hua Y., Keep R.F., Xiang J., Hoff J.T., Xi G. (2002). Thrombin-receptor activation and thrombin-induced brain tolerance. J. Cereb. Blood Flow Metab.

[15] Hua Y., Keep R.F., Hoff J.T., Xi G. (2003). Thrombin preconditioning attenuates brain edema induced by erythrocytes and iron. J. Cereb. Blood Flow Metab.

[16] Karabiyikoglu M., Hua Y., Keep R.F., Ennis S.R., Xi G. (2004). Intracerebral hirudin injection attenuates ischemic damage and neurologic deficits without altering local cerebral blood flow. J. Cereb. Blood Flow Metab.

[17] De Castro Ribeiro M., Badaut J., Price M., Meins M., Bogousslavsky J., Monard D., Hirt L. (2006). Thrombin in ischemic neuronal death. Exp. Neurol.

[18] Borsello T., Clarke P.G., Hirt L., Vercelli A., Repici M., Schorderet D.F., Bogousslavsky J., Bonny C. (2003). A peptide inhibitor of c-Jun *N*-terminal kinase protects against excitotoxicity and cerebral ischemia. Nat. Med.

[19] Wiegler K., Bonny C., Coquoz D., Hirt L. (2008). The JNK inhibitor XG-102 protects from ischemic damage with delayed intravenous administration also in the presence of recombinant tissue plasminogen activator. Cerebrovasc. Dis.

[20] Hirt L., Badaut J., Thevenet J., Granziera C., Regli L., Maurer F., Bonny C., Bogousslavsky J. (2004). D-JNKI1, a cell-penetrating c-Jun-*N*-terminal kinase inhibitor, protects against cell death in severe cerebral ischemia. Stroke.

[21] Hoffmann M.C., Nitsch C., Scotti A.L., Reinhard E., Monard D. (1992). The prolonged presence of glia-derived nexin, an endogenous protease inhibitor, in the hippocampus after ischemia-induced delayed neuronal death. Neuroscience.

[22] Nitsch C., Scotti A.L., Monard D., Heim C., Sontag K.H. (1993). The glia-derived protease nexin 1 persists for over 1 year in rat brain areas selectively lesioned by transient global ischaemia. Eur. J. Neurosci.

[23] Wu H., Zhao R., Qi J., Cong Y., Wang D., Liu T., Gu Y., Ban X., Huang Q. (2008). The expression and the role of protease nexin-1 on brain edema after intracerebral hemorrhage. J. Neurol. Sci.

[24] Luthi A., Van der Putten H., Botteri F.M., Mansuy I.M., Meins M., Frey U., Sansig G., Portet C., Schmutz M., Schroder M. (1997). Endogenous serine protease inhibitor modulates epileptic activity and hippocampal long-term potentiation. J. Neurosci.

[25] Kvajo M., Albrecht H., Meins M., Hengst U., Troncoso E., Lefort S., Kiss J.Z., Petersen C.C., Monard D. (2004). Regulation of brain proteolytic activity is necessary for the *in vivo* function of NMDA receptors. J. Neurosci.

[26] Dihanich M., Kaser M., Reinhard E., Cunningham D., Monard D. (1991). Prothrombin mRNA is expressed by cells of the nervous system. Neuron.

[27] Striggow F., Riek-Burchardt M., Kiesel A., Schmidt W., Henrich-Noack P., Breder J., Krug M., Reymann K.G., Reiser G. (2001). Four different types of protease-activated receptors are widely expressed in the brain and are up-regulated in hippocampus by severe ischemia. Eur. J. Neurosci.

[28] Ubl J.J., Vohringer C., Reiser G. (1998). Co-existence of two types of [Ca^2+^]i-inducing rotease-activated receptors (PAR-1 and PAR-2) in rat astrocytes and C6 glioma cells. Neuroscience.

[29] Choi B.H., Suzuki M., Kim T., Wagner S.L., Cunningham D.D. (1990). Protease nexin-1. Localization in the human brain suggests a protective role against extravasated serine proteases. Am. J. Pathol.

[30] Sokolova E., Reiser G. (2008). Prothrombin/thrombin and the thrombin receptors PAR-1 and PAR-4 in the brain: Localization, expression and participation in neurodegenerative diseases. Thromb. Haemost.

[31] Thevenet J., Angelillo-Scherrer A., Price M., Hirt L. (2009). Coagulation factor xa activates thrombin in ischemic neural tissue. J. Neurochem.

[32] Weinstein J.R., Gold S.J., Cunningham D.D., Gall C.M. (1995). Cellular localization of thrombin receptor mRNA in rat brain: Expression by mesencephalic dopaminergic neurons and codistribution with prothrombin mRNA. J. Neurosci.

[33] Hengst U., Albrecht H., Hess D., Monard D. (2001). The phosphatidylethanolamine-binding protein is the prototype of a novel family of serine protease inhibitors. J. Biol. Chem.

[34] Bonny C., Oberson A., Negri S., Sauser C., Schorderet D.F. (2001). Cell-permeable peptide inhibitors of JNK: Novel blockers of beta-cell death. Diabetes.

[35] Xi G., Reiser G., Keep R.F. (2003). The role of thrombin and thrombin receptors in ischemic, hemorrhagic and traumatic brain injury: Deleterious or protective?. J. Neurochem.

[36] De Castro Ribeiro M., Hirt L., Bogousslavsky J., Regli L., Badaut J. (2006). Time course of aquaporin expression after transient focal cerebral ischemia in mice. J. Neurosci. Res.

[37] Hirt L., Ternon B., Price M., Mastour N., Brunet J.F., Badaut J. (2009). Protective role of early aquaporin 4 induction against postischemic edema formation. J. Cereb. Blood Flow Metab.

[38] Donovan F.M., Pike C.J., Cotman C.W., Cunningham D.D. (1997). Thrombin induces apoptosis in cultured neurons and astrocytes via a pathway requiring tyrosine kinase and RhoA activities. J. Neurosci.

[39] Pike C.J., Vaughan P.J., Cunningham D.D., Cotman C.W. (1996). Thrombin attenuates neuronal cell death and modulates astrocyte reactivity induced by beta-amyloid *in vitro*. J. Neurochem.

[40] Vaughan P.J., Pike C.J., Cotman C.W., Cunningham D.D. (1995). Thrombin receptor activation protects neurons and astrocytes from cell death produced by environmental insults. J. Neurosci.

[41] Wang Y., Luo W., Reiser G. (2007). The role of calcium in protease-activated receptor-induced secretion of chemokine GRO/CINC-1 in rat brain astrocytes. J. Neurochem.

[42] Wang Y., Luo W., Reiser G. (2007). Proteinase-activated receptor-1 and -2 induce the release of chemokine GRO/CINC-1 from rat astrocytes via differential activation of JNK isoforms, evoking multiple protective pathways in brain. Biochem. J.

[43] Wang Y., Luo W., Stricker R., Reiser G. (2006). Protease-activated receptor-1 protects rat astrocytes from apoptotic cell death via JNK-mediated release of the chemokine GRO/CINC-1. J. Neurochem.

[44] Badaut J., Hirt L., Price M., de Castro Ribeiro M., Magistretti P.J., Regli L. (2005). Hypoxia/hypoglycemia preconditioning prevents the loss of functional electrical activity in organotypic slice cultures. Brain Res.

[45] Murer V., Spetz J.F., Hengst U., Altrogge L.M., de A.A., Monard D. (2001). Male fertility defects in mice lacking the serine protease inhibitor protease nexin-1. Proc. Natl. Acad. Sci. USA.

[46] Sinnreich M., Meins M., Niclou S.P., Suidan H.S., Monard D. (2004). Prothrombin overexpressed in post-natal neurones requires blood factors for activation in the mouse brain. J. Neurochem.

